# Strengthening immunisation supply chain systems through the GAVI Alliance Immunisation Supply Chain Strategy

**DOI:** 10.1186/2052-3211-7-S1-O7

**Published:** 2014-12-17

**Authors:** Kaleb Brownlow, Daniel Thornton

**Affiliations:** 1 GAVI Alliance, Geneva, Switzerland

## Background

As countries expand immunisation programmes to include new vaccines and ensure increased coverage and equity, existing and potential constraints exist within the supply chain systems to manage an estimated four-fold increase in vaccine volume per fully immunised child and a five-fold increase in the cost of vaccines to fully vaccinate a child from 2010 to 2020. To address existing and anticipated future supply chain system challenges, the Alliance has developed a comprehensive strategy.

## Method

The Alliance used a collaborative governance structure, drew upon extensive consultations, and priority working groups to develop the strategy. First, a governance structure included a Steering Committee comprised of Alliance leadership and a Task Force co-chaired by UNICEF and the GAVI Secretariat. Second, extensive consultations from countries, the global health community, Alliance and non-traditional partners via face-to-face meetings, forums, and informant interviews provided critical inputs. Third, working groups drew upon the work of experts and practitioners.

## Results

The GAVI Alliance Board has approved a comprehensive strategy that envisions that by 2020, all countries will have an immunisation supply chain system that provide potent vaccines efficiently to all with the ultimate goal to save children’s lives and protect people’s health by increasing access to immunisation in poor countries. Five pillars support the vision: people & practice, cold chain equipment, data for management, distribution and system design. Expected benefits for countries include improved ability to reach more people, strengthened leadership and human resources, and access to financial and technical resources to improve health supply chain systems.

## Discussion

The strategy focuses on ensuring fundamentals are in-place for each country, referred to as the 3+1 Approach that focuses on Supply Chain Managers, Supply Chain Management and Improvement Plans, Performance Dashboards, and Supply Chain System Design. Further, the strategy encourages countries to consider convergence between immunisation supply chains and those for other health commodities, and private sector and non-governmental partnerships where appropriate. This convergence will contribute to improved effectiveness and/or efficiency. To realise expected benefits and implement the 3+1 Approach, countries will need to commit greater human resources and financing, institutionalise accountability and performance, and be willing to test new approaches and systems. The Alliance is bringing additional resources to support these improvements.

## Lessons learned

Collaborative structures enabled the Alliance partners to bring varied perspectives, expertise, and capacity in supply chain systems to ensure organisational commitment to the strategy and a comprehensive strategy that addresses people, data, technology and equipment, and process.

